# FIB-4 Index and Neutrophil-to-Lymphocyte-Ratio as Death Predictor in Coronary Artery Disease Patients

**DOI:** 10.3390/biomedicines11010076

**Published:** 2022-12-28

**Authors:** Melania Gaggini, Fabrizio Minichilli, Francesca Gorini, Serena Del Turco, Patrizia Landi, Alessandro Pingitore, Cristina Vassalle

**Affiliations:** 1 Institute of Clinical Physiology, National Research Council, 56124 Pisa, Italy; 2 Fondazione Gabriele Monasterio, CNR-Regione Toscana, 56124 Pisa, Italy

**Keywords:** fibrosis-4 index, FIB-4, neutrophil-to-lymphocyte-ratio, NLR, hepatic fibrosis, systemic inflammation, biomarkers, coronary artery disease, outcome, mortality

## Abstract

Nonalcoholic fatty liver disease (NAFLD)-associated liver fibrosis is likely related to coronary artery disease (CAD) by the mediation of systemic inflammation. This study aimed at evaluating the predictive value of neutrophil-to-lymphocyte-ratio (NLR) and fibrosis-4 index (FIB-4), indices of inflammation and fibrosis, respectively, on CAD mortality. Data from 1460 CAD patients (1151 males, age: 68 ± 10 years, mean ± SD) were retrospectively analyzed. Over a median follow-up of 26 months (interquartile range (IQR) 12–45), 94 deaths were recorded. Kaplan–Meier survival analysis revealed worse outcomes in patients with elevation of one or both biomarkers (FIB-4 > 3.25 or/and NLR > 2.04, log-rank *p*-value < 0.001). In multivariate Cox regression analysis, the elevation of one biomarker (NLR or FIB-4) still confers a significant independent risk for mortality (hazard ratio (HR) = 1.7, 95% confidence interval (95% CI): 1.1–2.7, *p* = 0.023), whereas an increase in both biomarkers confers a risk corresponding to HR = 3.5 (95% CI: 1.6–7.8, *p* = 0.002). Categorization of patients with elevated FIB-4/NLR could provide valuable information for risk stratification and reduction of residual risk in CAD patients.

## 1. Introduction

Coronary artery disease (CAD), the most common form of cardiovascular disease (CVD), indicates the reduction of blood flow (ischemia) to the heart caused by narrowing or blockage of coronary arteries, mainly due to atherosclerotic plaque onset and development. Nonalcoholic fatty liver disease (NAFLD) is a condition that includes a spectrum of liver alterations such as steatosis, nonalcoholic steatohepatitis (NASH), liver fibrosis, and other complications like cirrhosis and carcinoma [1]. A recent meta-analysis investigated the specific association between NAFLD and risk of CV events (including risk of myocardial infarction, ischemic stroke, atrial fibrillation, and heart failure), finding a significant association [2]. This result was confirmed by another recent work from the UK Biobank, evidencing NAFLD (defined using fatty liver index, FLI) as an independent predictor for all-cause mortality and adverse CVD outcomes (mortality, acute myocardial infarction, and stroke) [3]. In particular, NAFLD is common in patients having co-existing obesity, diabetes mellitus, or metabolic syndrome, all of which are established risk factors for CAD, also associated with the presence, severity, and outcomes of ischemic cardiovascular disease. Conversely, ischemic heart disease is considered the primary cause of death in NAFLD patients with advanced liver fibrosis [4].

Although liver biopsy is still the gold standard, simple calculated fibrosis biomarkers have been included in the current guidelines as tools helpful in reducing the need for liver biopsy and identifying fibrosis in clinical practice, with advantages in terms of simplicity and non-invasiveness. In particular, the fibrosis-4 index (FIB-4) was reported as a simple-to-use parameter to evaluate liver fibrosis, calculable through four easily available laboratory parameters: age, aspartate aminotransferase (AST), alanine aminotransferase (ALT), and platelet count. Recently, FIB-4, together with other fibrosis scores, was reported to be associated with increased risk of mortality among CAD patients, highlighting its potential as a prognostic biomarker in CAD [3]. Moreover, FIB-4 was also associated with increased risk of CV events (fatal or nonfatal ischemic stroke and myocardial infarction, cardiac or peripheral revascularization, atrial fibrillation and CV death) in a large cohort of patients (*n* = 898) screened for NAFLD and followed over a median follow-up time of 41.4 months [5]. 

Although NAFLD and ischemic CVD share common risk factors (e.g., dyslipidemia, insulin resistance, and endothelial dysfunction), these conditions are also closely linked through multiple pathophysiological mechanisms, including effects related to fat accumulation in the liver and inside and around the heart, hepatic/peripheral insulin resistance, and genetic and gut microbiota changes [3,4,6,7,8,9]. In particular, systemic inflammation appears as a key bridge connecting NAFLD with CAD, atherosclerosis and CAD being considered inflammatory conditions [1,6]. Together with common clinically used inflammatory parameters, various parameters easily obtainable from complete blood counts can be calculated, and have been recently proposed as markers of inflammatory status associated with various manifestations of ischemic CVD as well as NAFLD [10,11,12]. In particular, neutrophil-to-lymphocytes-ratio (NLR) combines neutrophils and lymphocytes, thus reflecting neutrophil action, which secretes inflammatory mediators and modulates inflammation by releasing myeloperoxidase and superoxide radicals, while lymphocytes represent the regulatory pathway of the immune system, as low lymphocyte counts are a common finding during a stress response [13]. The predictive power of NLR has been shown for adverse CV events [11,12,14]. Furthermore, NLR and other blood count indices (including platelets in their formulas) have been proven to be useful for assessing prognosis of patients with NAFLD and chronic myeloproliferative neoplasms, which are inflammatory neoplastic diseases with high CV burden [14,15].

Several studies have indicated that evaluating a combination of biomarkers from different pathophysiological pathways—a multimarker approach—could improve prognostic information (e.g., based on the number of elevated biomarkers by their cutoffs or in the assignment of a point-based score to each biomarker) when compared to the information provided by any single biomarker [16,17,18,19,20]. Since previous data demonstrated that patients with increased levels of FIB-4 or NLR have a significantly worse prognosis, we hypothesized that the simultaneous evaluation of two biomarkers such as FIB-4 (as an index of fibrosis) and NRL (as an index of inflammation) may provide complementary information and help improve predictive performance more effectively for risk stratification and reduction of residual risk in CAD, as assessed using Kaplan–Meier curve analysis and the Cox proportional hazard model. For this purpose, patients were divided into those for whom the two markers were below the cutoff value (two marker low group), patients in whom one marker was above the cutoff value, and the other one below the cutoff values (one marker high group) and patients in whom all two biomarkers were increased (two marker high group).

## 2. Materials and Methods

### 2.1. Characteristics of the Study Population

This was a retrospective single-center cohort study, including 1460 patients (1151 males, age: 68 ± 10 years, mean ± SD) with coronary angiographically proven CAD. All data used in this study were retrieved from the clinical record collected in the Image database, which stored data collected upon patient admission in the Cardiology Department of the Institute of Clinical Physiology-CNR in Pisa [21]. All data were acquired in the context of institutional assistance within clinical care purposes in a retrospectively collected modality from our Institution’s patient dataset (Image database), including clinical characteristics, previous clinical history, CAD risk factors, comorbidities, laboratory and instrumental results, pharmacological therapies, and post-discharge follow-up outcomes. All provided data are completely anonymous and were evaluated as aggregated, not individually. Patients presenting severe systemic diseases including neoplasia, acute or chronic inflammatory disease, immunological disease, or patients refusing or unable to supply written informed consent were not enrolled.

Data on smoking history, arterial hypertension (systolic blood pressure >140 mmHg/diastolic pressure >90 mmHg/use of antihypertensive medication), type 2 diabetes (T2D, fasting plasma glucose >126 mg/dL/antidiabetic treatment), obesity (body mass index, BMI above 30 kg/m^2^), and dyslipidemia (total cholesterol ≥200 mg/dL/triglyceride ≥150 mg/dL/use of lipid-lowering drugs) were coded in a dichotomized fashion. Medical therapy included angiotensin-converting enzyme inhibitors, beta-blockers, lipid-lowering agents, antidiabetic agents, diuretics, and aspirin.

This study was carried out in accordance with the Code of Ethics of the World Medical Association (Declaration of Helsinki) for experiments involving humans.

### 2.2. Blood Sampling

In all patients, venous peripheral blood samples were drawn on admission for laboratory measurement. Blood samples were stored at room temperature before the evaluation on automated routine analysis systems. NLR was calculated as neutrophil (N) to lymphocyte (L) ratio by complete blood count analysis, utilizing as cutoff the 50th percentile, corresponding to 2.04 [22].

For FIB-4, the score parameters used were age, platelet counts, and AST and ALT levels according to the following Formula: [age (years) × AST (U/L)]/[PLT (10^9^/L) × ALT(U/L) 1/2] and using >3.25 as the cutoff [23].

### 2.3. Follow-Up

The endpoint of this study was all-cause mortality. Follow-up was performed using telephone calls, personal communication with the patient’s physician, or outpatient control visits. Patients were followed from admission until the endpoint (mortality, death information derived from medical records, or death certificates) or up to 120 months from the time of enrollment. The definition of cardiac death required the following documentation: significant arrhythmias, cardiac arrest, death attributable to congestive heart failure, or myocardial infarction in the absence of any other precipitating factors.

### 2.4. Statistical Analysis

Continuous variables were presented as mean ± SD; categorical variables were presented as counts and percentages (%). Spearman’s rank correlation was used to measure the strength and direction of association between two datasets. Statistical analysis performed included the χ^2^ test for categorical variables, using the Statview statistical package, version 5.0.1 (SAS Institute Inc., Cary, NC, USA). Logistic analysis was applied to predict the association of independent variables with one dichotomous dependent variable (FIB-4 or NLR).

The survival analysis was performed using the Kaplan–Meier method with the log-rank test. The multivariate Cox proportional hazard regression was used to evaluate the effect of variables on survival time, yielding data as hazard ratio (HR) with a 95% confidence interval (95% CI). Variables were included in the multivariate Cox model based on significance of the univariate analyses. Statistical significance was set at a two-sided *p*-value < 0.05.

## 3. Results

### 3.1. Characteristics of the Studied Population

The study population included 1460 angiographically proven CAD patients, 1151 (79%) men and 309 women (21%). The mean age was 68 years. The characteristics of the CAD cohort according to FIB-4 (cutoff 3.25) and NLR (50th percentile corresponding to 2.04) are reported in Table 1, with data expressed as a number (percentage, as a proportion with respect to the total number of the considered subgroup). Patients with higher FIB-4 were characterized by a significantly higher proportion of older subjects, reduced left ventricular ejection fraction (LVEF), and higher NLR (>3.25), and a significantly lower percentage of patients with dyslipidemia (treated with lipid-lowering drugs or not). Moreover, patients with higher NLR (<2.04) included a significantly higher percentage of older subjects, with reduced LVEF and higher FIB-4, and a significantly lower proportion of individuals with dyslipidemia (treated with lipid-lowering drugs or not).

### 3.2. Spearman’s Rank Correlation between NLR and FIB-4 and the Variables Included in the Calculation of the Two Indexes

Spearman’s rank correlation tests showed a significant correlation between NLR and FIB-4 (z-value 4.22; *p* < 0.001) and between N and age (z-value 3.62; *p* < 0.001) and AST (z-value 4.1; *p* < 0.001), but not with ALT (z-value 1.8; *p* = 0.07) or PLT (z-value 1.5; *p* = 0.15), and between L and age (z-value −5.5; *p* < 0.001) and AST (z-value −4.1; *p* < 0.001), but not ALT (z-value −1.34; *p* = 0.18) and PLT (z value −0.6; *p* = 0.55).

### 3.3. Unadjusted and Adjusted Logistic Analysis

From the variables listed in Table 1, age (>69 years), dyslipidemia, LVEF (<50%), and NLR (>2.04) were significant determinants for elevated FIB-4 (>3.25) in the univariate logistic analysis. After multivariate adjustment, NLR (HR = 1.8, 95% CI: 1.1–3.1, *p* = 0.019) and aging (HR = 2.4, 95% CI: 1.4–4.1, *p* = 0.0012) remained as independent predictors for elevated FIB-4.

From the variables listed in Table 1, age (>69 years), dyslipidemia, LVEF (<50%), FIB-4 (>3.25), and multivessel disease were significant determinants for increased NLR in the univariate logistic analysis. In the logistic regression analysis, reduced EF (<50%, HR = 1.8, 95% CI: 1.4–2.2, *p* < 0.001), dyslipidemia (HR = 0.6, 95% CI: 0.5–0.9, *p* = 0.009) and elevated FIB-4 (HR = 2, 95% CI: 1.2–3.3, *p* = 0.019) remained independent predictors for higher NLR.

### 3.4. Follow-Up

**Figure 1:**
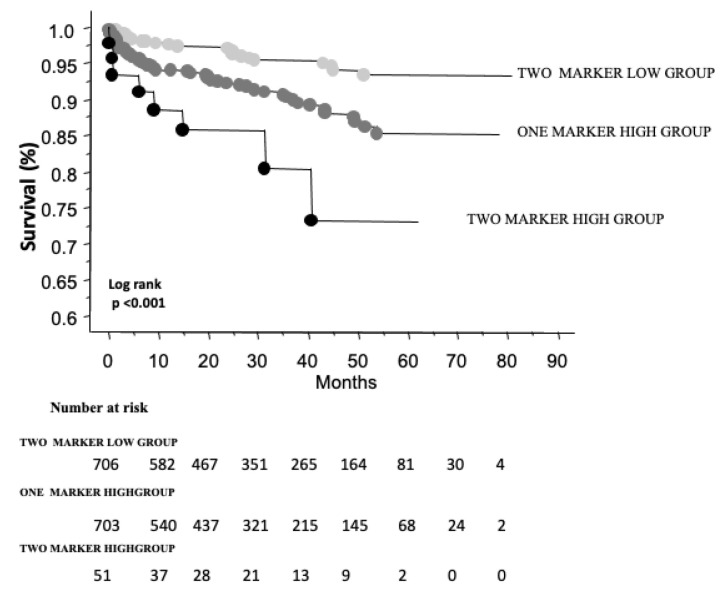
Overall survival stratified by Kaplan–Meier survival curves according to FIB-4 and NLR categories.

Over a median follow-up time of 26 months (interquartile range, IQR: 12–45), a total of 94 deaths were recorded. Kaplan–Meier survival analysis showed worse survival among patients in the both marker or one marker high groups, compared with those in the two-marker low group (number of events/total number 8/51, 15.7%; 59/703, 8.4%, versus 27/706, 3.8%, respectively, in the three groups). An intermediate risk was observed in those with an increase in one of the two biomarkers (FIB-4 or NLR), and a worse outcome in those in the two biomarker high group, when compared with patients in the two marker low group (Figure 1).

In Table 2, results from the univariate Cox analysis are shown for all variables reported in Table 1, also considering the parameters “one marker high group” and “two marker high group”. In particular, dyslipidemia (HR = 0.35, 95% CI: 0.22–053, *p* < 0.001), obesity (BMI > 30 kg/m^2^; HR = 0.46, 95% CI: 0.24–0.86, *p* = 0.015), age (>69 years; HR = 3.7, 95% CI: 2.3–6.1, <0.001), and LVEF (<50%; HR = 2.4, 95% CI: 1.6–3.7, *p* < 0.001) were predictors for mortality in the univariate Cox analysis. Moreover, the increase in one of the two biomarkers (FIB-4 or NLR) confers a risk for mortality of 2.4, 95% CI: 1.5–3.7 *p* < 0.001, while elevation of both biomarkers (FIB-4 and NLR) a risk of 5.0 (95% CI: 2.2–11.0, *p* < 0.001).

**Figure 2:**
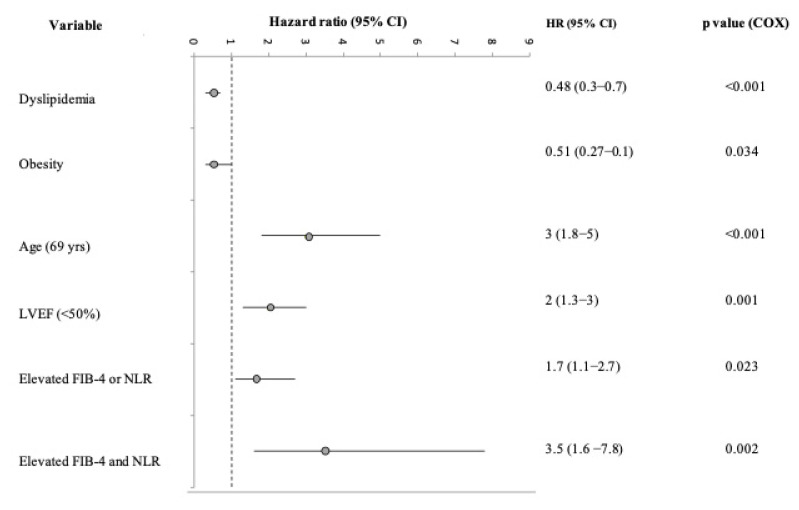
Independent predictors (hazard ratios; 95% CI, *p* value) for mortality in multivariate Cox analysis, including parameters that are significant determinants in the univariate analysis along with single or combined presence of elevated FIB-4 and NLR. Dyslipidemia = total cholesterol ≥200 mg/dL/triglyceride ≥150 mg/dL/use of lipid-lowering drugs. Obesity = body mass index above 30 kg/m^2^. Abbreviations: LVEF = left ventricular ejection fraction.

After adjustment for all variables significantly associated with mortality in the univariate analysis, the multivariate Cox regression model showed that the presence of one biomarker elevation still confers a significant independent risk of developing mortality (HR = 1.7, 95% CI: 1.1–2.7, *p* < 0.023), whereas an increase in both biomarkers confers a significant independent risk of 3.5 (95% CI: 1.6–7.8, *p* < 0.002) (Figure 2). In addition, the presence of dyslipidemia or obesity was associated with improved survival (HR < 1), while older age and reduced LVEF (HR > 1) suggested an increased risk and as such were associated with reduced survival.

Moreover, the trend of mortality risk for unit increment of the biomarkers was also performed, giving HR = 1.13, 95% CI: 1.05–1.22, *p* < 0.001, and HR = 1.11, 95% CI: 1.07–1.15, *p* < 0.001 for FIB-4 and NLR, respectively, in the univariate analysis. Moreover, multivariate Cox analyses including continuous NLR and FIB-4 parameters were performed, giving HR = 1.08, 95% CI: 1–12, *p* = 0.13, and HR = 1.09, 95% CI: 1.05–1.13, *p* < 0.001 for FIB-4 and NLR, respectively, after adjustment for dyslipidemia, obesity, aging, and reduced LVEF. We also tested the continuous parameter “FIB-4+NLR”, which was normalized in a 0–100 scale as ((FIB-4+NLR–min (FIB-4+NLR))/(max (FIB-4+NLR)–min (FIB-4+NLR))) ∗ 100, using this composite parameter for univariate and multivariate analysis for mortality. In univariate analysis an HR of 1.04 was obtained (95% CI: 1.02–1.06, *p* < 0.001) for FIB-4+NLR. After adjustment in multivariate analysis for dyslipidemia, obesity, aging, and reduced LVEF, an HR of 1.03 (95% CI: 1.02–1.05, *p* < 0.001) was obtained for the variable FIB-4+NLR.

For all Cox regression models, the assumption of proportional risk was respected.

## 4. Discussion

The presence of elevated FIB-4 or/and NLR is associated with worse clinical outcomes in CAD patients. Thus, the categorization of patients with elevated FIB-4 or/and NLR could provide valuable information for risk stratification and reduction of residual risk in CAD patients.

NAFLD is a chronic liver disease whose progression appears closely related to ischemic CAD [24]. Although the specific underlying common pathogenetic mechanisms are not fully elucidated, metabolic syndrome could act as a key determinant, causing organ damage in both the heart and liver. Indeed, patients with NAFLD generally are overweight/obese and may present with insulin resistance/T2D, dyslipidemia, or hypertension, all of these factors being components of metabolic syndrome and at the same time well-known CVD risk factors [25]. Accordingly, many data suggest that the presence of NAFLD is associated with an increasing prevalence and incidence of CAD [26,27]. Therefore, it is reasonable to hypothesize that NAFLD evaluation could improve CV risk assessment in the general population as well [28,29].

However, current score systems actually recommended by guidelines for assessing CVD risk in asymptomatic adults (e.g., Framingham Risk Score, European SCORE-2 and SCORE-2 OP, the ASCVD score), although based on the identification of several risk factors, may fail to correctly identify NAFLD-related CVD because parameters such as insulin resistance are not included in their calculation [25]. Thus, NAFLD could be added to current scoring systems for the prediction of CVD. Additionally, some results (though not all) have indicated a relationship between progression of NAFLD and CAD outcomes. In particular, a meta-analysis (16 studies, 34,043 individuals, 36.3% with NAFLD and approximately 2600 CVD outcomes, >70% CVD deaths, with a median follow-up of 6.9 years) reported a significant association between NAFLD and an increased risk of fatal and non-fatal CV events (odds ratio 2.58, 95% CI: 1.78–3.75) [30]. However, the observational nature of the studies evaluated still fails to demonstrate definitive causality of NAFLD for CVD. A more recent meta-analysis conducted on a very large adult population (36 longitudinal studies, 5,802,226 middle-aged individuals, mean age 53 ± 7 years, 99,668 incident cases of fatal and non-fatal CVD events, median follow-up of 6.5 years) reported a significant association between NAFLD and an increased long-term risk of fatal or non-fatal CVD events (pooled HR = 1.45, 95% CI: 1.31–1.61) [31]. Interestingly, CVD risk is further increased with more advanced liver disease, especially in subjects with higher fibrosis stages. Conversely, another meta-analysis (14 studies, 498,501 subjects, 24,234 deaths) of patients with NAFLD evidenced that this condition represents a predictor of increased all-cause mortality (HR = 1.34, 95% CI: 1.17–1.54) but not CVD or cancer mortality [32]. Of note, a further meta-analysis conducted on hospitalized CVD patients with NAFLD revealed a significantly higher risk of all-cause mortality than non-NAFLD patients (adjusted HR = 2.08, 95% CI: 1.56–2.59, *p* < 0.001), identifying these patients as those who may greatly benefit from NAFLD assessment in their clinical management [33].

Although liver biopsy remains the “gold standard” tool for the diagnosis and quantification of NAFLD, it is an invasive method with potential complications; non-invasive tests (e.g., clinical scores, biochemical biomarkers, and liver elastography) may represent reliable alternative tools for the diagnosis and staging of NAFLD. In particular, European guidelines recommend the use of clinical scores in all patients with NAFLD [34], while the current international guidelines for hepatitis C treatment recommend the use of laboratory indices to assess the extent of hepatic fibrosis [35,36]. Furthermore, some findings supported their utility in identifying subjects at high risk of severe liver disease [37]. Notably, given its simplicity, availability, and reliability, the FIB-4 index has been proposed as an alternative tool to stratify the risk of liver-related outcomes in NAFLD [38] and has also been found to be associated with increased risk of CV events [5]. Additionally, a very recent meta-analysis (12 studies, 25,252 patients with CVD) showed a significant association between the highest baseline level of FIB-4 and an increased risk of CV events (HR = 1.75, 95% CI: 1.53–2.00), CV mortality (HR = 2.07, 95% CI: 1.19–3.61) and all-cause mortality in patients with CVD (HR = 1.81, 95% CI: 1.24–2.66) [39].

NLR is a biomarker of systemic inflammation that can be calculated from a simple blood count, and therefore provides cheap, easily obtainable, and widely available information in clinical practice. This biomarker integrates two important immune pathways: neutrophils for persistent inflammation, which can facilitate plaque disruption, and lymphocytes for the regulation of inflammatory response and an anti-atherosclerotic effect [40,41,42]. Consistently, this biomarker has been correlated with different inflammatory and cardiometabolic diseases, resulting in an inexpensive, widely available and reliable tool to improve risk stratification and predict major adverse events in patients with different cardiometabolic conditions [12,22,40,43,44,45,46].

The predictive power of NLR in patients with liver fibrosis and liver cirrhosis has also been reported [47]. Moreover, some recent data have highlighted how FIB-4 is independently associated with the presence and severity of CAD after adjustment for traditional risk factors, as well as the role of NLR as a mediator of the relationship between NAFLD fibrosis and CAD severity [48]. We further complete this information, showing that the presence of high levels of one or both these biomarkers is associated with increased risk of major adverse events in CAD patients. Importantly, although NLR and FIB-4 might be considered two biomarkers that allegedly stand for two different pathways and pathophysiological phenomena, and as such reflect different levels of information, the Spearman’s rank correlation test showed a significant correlation between NLR and FIB-4, which gives proof of verification based on the concept that liver fibrosis is closely related to hepatic and systemic inflammation.

We also observed a significant inverse relationship between higher NLR and lower LVEF in stable CAD, which suggests that this simple and widely available parameter may represent a useful predictor of impaired LV function in a CV setting. Notably, NLR has been associated with reduced LVEF in acute myocardial infarction, and its correlation with dROMS (a marker of oxidative stress) in an asymptomatic population emphasizes the interconnected association between oxidative stress and inflammatory burden, of which NLR may represent a reflection [49,50,51].

In Cox regression models, dyslipidemia appeared as a very strong protective factor for post-CAD mortality, a result that may surprise. Nonetheless, it should be considered that the definition of dyslipidemia, as reported in the materials and methods section, included patients under treatment with lipid-lowering drugs (mainly statins). It is well known that statin therapy is associated with a lower risk of all-cause mortality in coronary artery disease patients, likely due to their immunomodulatory, anti-inflammatory, and anti-atherosclerotic effects, possibly contributing to our finding [51,52,53,54]. This also applies to obesity, which, as it is a well-established independent risk factor for the development of many cardiovascular conditions (e.g., heart failure, coronary heart disease, atrial fibrillation, and hypertension), it is logical to expect it to have a close association with mortality. However, a large body of evidence points to an obesity paradox, with a better prognosis for obese patients than lean patients with an identical CV burden [55]. This is because adipose tissue is not inert, but an endocrine plastic element also capable of releasing protective molecules (e.g., adipokines and other bioactive molecules), whose total effect essentially depends on the balance between beneficial effects important for the metabolic homeostasis of the whole body, while dysfunctional processes may contribute to the pathogenesis of cardiometabolic disease [56].

## 5. Study Strengths and Limitations

This is a large retrospective study, and the parameters evaluated were calculated using inexpensive and readily available laboratory measurements in the context of major adverse CV events in patients with stable CAD. However, the study also retains some limitations related to its retrospective nature and the single-center experience, which require further confirmation of results in future prospective multicenter trials including large-scale cohorts. Additionally, our results were based on a single blood sampling, while inter- and intra-individual variations in blood count-based indices over time are possible. It should also be considered that FIB-4 and NLR are surrogate markers of the processes it is hypothesized are responsible for the relationship observed.

Other cardiovascular conditions that may be associated with NAFLD (e.g., atrial fibrillation, history of stroke) were not available in the database. These are important factors to be considered in future studies, and also to adjust for in the regression models, as these may act as confounders on the association explored. Moreover, the effect of specific drugs needs to be further explored, considering parameters that were not available in our database (e.g., types of drugs, drug delivery, doses, and treatment time duration).

## 6. Conclusions

CAD patients showing elevated FIB-4 or/and NLR were more likely to die. Categorization of patients with elevated FIB-4/NLR could therefore provide valuable information for risk stratification and reduction of residual risk in CAD patients. A heart–liver team would be encouraged to further reveal the complex molecular and cellular network linking NAFLD to CAD and verify whether lifestyle modification and disease-targeted drug approaches may benefit both conditions.

## Figures and Tables

**Table 1:** Demographic and clinical characteristics of the studied cohort according to FIB-4 and NLR levels.

Parameter	FIB-4			NLR		
	≤3.25	>3.25	*p*	≤2.04	>2.04	*p*
	*n* = 1385	*n* = 75		*n* = 730	*n* = 730	
Age (years)	68 ± 10	74 ± 9	<0.001	67 ± 9	69 ± 10	<0.001
Age (<69 years)	700 (50)	55 (73)	<0.001	336 (46)	419 (57)	<0.001
Male sex	1097 (79)	54 (72)	ns	582 (51)	569 (49)	ns
Body mass index (kg/m^2^) (*n* = 1429)	27 ± 4	27 ± 4	ns	28 ± 4	27 ± 4	0.014
Obesity	306 (22)	20 (27)	ns	173 (24)	153 (24)	ns
Hypertension	831 (60)	42 (56)	ns	442 (61)	431 (59)	ns
Diabetes	431 (31)	31 (41)	ns	225 (31)	237(32)	ns
Dyslipidemia/lipid-lowering drugs	1172 (85)	56 (75)	0.022	638 (87)	590 (81)	<0.001
Smoking habit (current/former smokers)	646 (47)	30 (40)	ns	341 (47)	335 (46)	ns
LVEF (%) (*n* = 1448)	52 ± 11	48 ± 12	<0.001	53 ± 10	50 ± 4	<0.0001
LVEF (<50%)	368 (26)	29 (39)	<0.22	154 (21)	243 (33)	<0.001
Multivessel disease	847 (61)	54 (72)	ns	434 (59)	467 (64)	ns
FIB-4	-	-	-	1.54 ± 0.76	1.88 ± 1.85	<0.001
FIB-4 (>3.25)	-	-	-	24 (3)	51 (7)	0.0014
NLR	2.57 ± 2.4	4.9 ± 5.39	<0.001	-	-	-
NLR (>2.04)	679 (49)	51 (68)	0.0014	-	-	-

Data are expressed as mean ± SD or number (%). Hypertension = systolic blood pressure >140 mmHg/diastolic pressure >90 mmHg/use of antihypertensive medication. Diabetes = fasting plasma glucose >126 mg/dL twice/antidiabetic treatment. Dyslipidemia = total cholesterol ≥200 mg/dL/triglyceride ≥150 mg/dL/use of lipid-lowering drugs. Obesity = body mass index above 30 kg/m^2^. Abbreviations: LVEF = left ventricular ejection fraction. ns = not statistically significant.

**Table 2:** Univariate Cox analysis for mortality.

Parameter	Mortality		
	Hazard Ratio	95% CI	*p*
Age (years)	1.08	1.05–1.11	<0.001
Age (<69 years)	3.7	2.3–6.1	<0.001
Male sex	0.96	0.6–1.6	ns
Body mass index (kg/m^2^) (*n* = 1429)	0.87	0.8–0.9	<0.001
Obesity	0.46	0.24–0.86	0.015
Hypertension	0.85	0.6–1.3	ns
Diabetes	1.13	0.7–1.7	ns
Dyslipidemia/lipid-lowering drugs	0.35	0.22–0.53	<0.001
Smoking habit (current/former smokers)	1.1	0.7–1.7	ns
LVEF (*n* = 1448)	0.96	0.94–0.97	<0.001
LVEF (<50%)	2.4	1.6–3.7	<0.001
Multivessel disease	1.4	1–2.1	ns
FIB-4	1.13	1.05–1.22	0.0011
FIB-4 (>3.25)	2.35	1.22–4.52	0.011
NLR	1.1	1.1–1.2	<0.001
NLR (>2.04)	2.4	1.6–3.8	<0.001
One marker high group	2.4	1.5–3.7	<0.001
Two marker high group	5	2.3–11	<0.001

Hypertension = systolic blood pressure >140 mmHg/diastolic pressure >90 mmHg/use of antihypertensive medication. Diabetes = fasting plasma glucose >126 mg/dL twice/antidiabetic treatment. Dyslipidemia = total cholesterol ≥200 mg/dL/triglyceride ≥150 mg/dL/use of lipid-lowering drugs. Obesity = body mass index above 30 kg/m^2^. Abbreviations: CI = confidence interval; LVEF = left ventricular ejection fraction. ns = not statistically significant.

## Data Availability

The data that support the findings of this study are available upon reasonable request (e.g., research purpose) from the authors.
